# Exploratory research: Is forensic science a cause of miscarriage of justice in Canada?

**DOI:** 10.1016/j.fsisyn.2026.100685

**Published:** 2026-05-09

**Authors:** Sara-Jane Lécuyer, Keleiali'i Urima, Carole Sénéchal, Frank Crispino

**Affiliations:** a Department of Biochemistry, Chemistry, Physics and Forensic Science, Université du Québec à Trois-Rivières (UQTR), 3351 boulevard des Forges, Trois-Rivières, Québec, G8Z 4M3, Canada; b Faculty of Education, University of Ottawa, 145 Jean-Jacques-Lussier, Ottawa, Ontario, K1N 6N5, Canada; c Groupe de Recherche en Science Forensique (GRSF), Université du Québec à Trois-Rivières, Pavillon CIPP, 3351 boulevard des Forges, Trois-Rivières, Québec, G8Z 4M3, Canada; d Centre International de Criminologie Comparée (CICC), Université du Québec à Trois-Rivières, 3351 Boulevard des Forges, Trois-Rivières, QC, G8Z 4M3, Canada

**Keywords:** Wrongful conviction, Miscarriage of justice, Forensic science, Expert evidence, Scientific errors

## Abstract

This exploratory research examines the implication of science in 92 Canadian wrongful convictions officially recognized by the state. The analysis revealed that science played a role in 30 cases, but 97% of those errors stemmed from the misinterpretation or overstatement of reliable scientific results, predominantly in legal medicine, and not from analytical errors. These errors often involved exceeding the probative value of evidence or a confusion between levels of interpretation.

In addition, a collaboration with AVANE (Aide aux Victimes des Affaires Non-Élucidés) a French organization providing support and assistance to unsolved cases, allowed to study three cold cases. Despite the small number of cases, all three of them showed an underestimation of the potential of the trace, the ontological nature of scientific evidence and its potential to generate alternative hypotheses to those resulting from investigative tunnel vision or technical and financial constraints. Hence, this exploratory research raises the hypothesis of systemic vulnerabilities: an overestimation of expertise for wrongful convictions (type I errors) and an underestimation of the potential of the trace for unsolved crimes (type II errors). Both types of errors could support a lack of understanding of the trace, its potential, and its limits. Should this exploratory study be confirmed, it would emphasize the need for a stronger forensic oversight all along the investigative process, improved scientific literacy among judicial actors, and frameworks, such as the one proposed by the Sydney Declaration, to better address the inherent complexity and uncertainty of forensic evidence.

## 1 Introduction and theoretical context

Forensic science, as part of the justice system, has often been criticized for its reliability and has even been blamed for miscarriages of justice by numerous authors [[1], [2], [3]]. Although the denunciation of the involvement of forensic science in wrongful conviction cases has gained momentum in recent years, the issue itself is far from new. At the beginning of the twentieth century, the Dreyfus Affair already illustrated the potential abuses of scientific evidence within the judicial system [4]. Alfred Dreyfus, a Jewish captain in the French army at the end of the 19th century, was wrongfully convicted of treason based on a handwriting analysis carried out on a document known as ‘Le Bordereau’ [5]. It later appeared that this analysis was flawed and biased. Rather than assessing the authorship of the text, the experts sought to prove Dreyfus's guilt, and contradictory evidence were reinterpreted to support this conclusion [5]. The involvement of Alphonse Bertillon further illustrates how preconceived beliefs, particularly anti-semitic bias, shaped the interpretation of the evidence [6].

This case highlights a central issue in forensic science, errors can stem not only from analytical limitation, but also from the interpretation and contextualisation of scientific results.

However, it is in recent decades that an extensive body of scientific research has emerged, casting serious doubt on the validity of many forensic disciplines and demonstrating the limited capacity of courts to safeguard their scientific credibility.

In the United States, forensic science was labelled as “junk science” by Huber in his 1991 book *Galileo's Revenge: Junk Science in the Courtroom* [2], a term used again 32 years later by Fabricant, a partner at the Innocence Project, in *Junk Science and the American Criminal Justice System* [1]. These criticisms are reminiscent of the findings of the National Academy of Sciences report, *Strengthening Forensic Science in the United States: A Path Forward (NAS report)* [7]. The report concluded that most forensic disciplines lack rigorously validated and universally accepted scientific practices, suffer from insufficient peer review, and tend to overestimate the probative value of individualization claims [7]. DNA analysis was an exception to the criticisms raised in the NAS report; however, even this “gold standard” of forensic evidence was later tempered by the *President's Committee of Advisors on Science and Technology* in its report *Forensic Science in Criminal Courts: Ensuring Scientific Validity of Feature-Comparison Methods* [8].

Following the NAS report, the majority of the literature has focused on the role of cognitive biases and the inferential aspects of interpretation mistakes in forensic science [7,[9], [10], [11]], while recognizing the importance of contextualising scientific evidence [[12], [13], [14], [15]]. Although this study does not focus primarily on cognitive biases, it is important to recognize that a large body of research has demonstrated their influence on forensic interpretation. Biases can affect the way in which evidence is selected, analyzed and interpreted, therefore contributing to errors in forensic reasoning. However, the aim of this study is to move beyond an approach focused exclusively on individual cognitive biases and to examine the broader, more systemic conditions that shape the use and interpretation of forensic science within the justice system.

More recent work calls for a deeper understanding of the cognitive and organisational processes that underlie forensic practice, emphasizing that errors may stem not only from the individual level, but also from broader systemic, institutional and cultural conditions [[16], [17], [18], [19], [20]]. This perspective is reflected in frameworks such as the Sydney Declaration [16], in which a consortium of experts sets out the fundamental principles of forensic science, refocusing the discipline on its core element: the trace. This study adopts this perspective by recognizing the systemic dimensions of context, rather than solely on individual cognitive biases, to better identify the structural factors likely to contribute to miscarriages of justice.

While not a discovery, the involvement of forensic science in wrongful convictions is not confined to the United States. Several reports, including one from the Public Prosecution Service of Canada, identify recurring causes of these errors, notably errors in scientific expertise and the difficulties faced by judicial actors in interpreting forensic evidence [21]. In *Justice Miscarried: Inside Wrongful Convictions in Canada*, Katz examines ten cases of wrongful convictions in Canada that occurred between 1968 and 1997 [22]. Innocence Canada, a legal aid and advocacy organization supporting individuals claiming to be victims of wrongful convictions, reports having contributed to the exoneration of 30 Canadians since 1993 [23], primarily through advances in DNA analysis [24]. Moreover, new forensic technological tools, such as forensic genealogy, have contributed to the resolution of cold cases [25].

In his book *De la police scientifique à la traçologie: le renseignement par la trace* (“From Forensic Science to Traceology: Intelligence from the trace”), Ribaux [26] distinguishes two categories of failure of justice:

- The first type of failure occurs when an innocent person is wrongfully convicted. These are analogous to false positives or type I errors.
- The second type of error refers to cold cases or situations in which the perpetrator is not convicted. This corresponds to a false negative or type II error.

Quantifying these two types of errors remains complex. Wrongful convictions (type I) can be identified and studied since they are officially recognized by the state. They are documented through the long and complex process of judicial exoneration and recorded by various organizations. It remains, however, impossible to quantify the number of people still unjustly incarcerated or convicted without official recognition of the error. Nonetheless, such cases are typically the ones covered by the media and in the literature. The literature, however, recognizes that wrongful convictions are complex and multifactorial phenomena that may involve biases, racial prejudice, erroneous eyewitness identification, reliance on police informants, and flawed scientific evidence [1,22,27].

Type II errors are even more difficult to detect, as they include unsolved crimes (cold cases) as well as crimes that were never detected by authorities and therefore never investigated. In criminology, this number is referred to as the “dark figure of crime” [28]. In such cases, miscarriages of justice may go entirely unnoticed. With the exception of wrongful convictions for crimes that never actually occurred, a type I error automatically causes a type II error, as convicting the wrong person leaves the crime unresolved until his recognized exoneration.

After reviewing and analyzing 92 cases of wrongful convictions in Canada, this exploratory study aims to question the failures attributed to forensic sciences, attempting also a look at type II errors. Are forensic errors the result of isolated human error, or could they stem from deeper systemic deficiencies?

## 2 Methodology

For type I errors, the research adopts a comparative methodological approach based on the literature, analyzed through the lens of the Sydney Declaration, which provides a conceptual framework that helps clarify the fundamental concepts of forensic science [16]. The sources include *Justice Miscarried: Inside Wrongful Convictions in Canada* [22], The Canadian Registry of Wrongful Convictions [29] and Innocence Canada [23]. Cases selected in this study met two criteria: (1) the individual had been convicted by a Canadian court, and (2) the conviction was later officially recognized as wrongful by a provincial or federal court.

A total of 92 Canadian wrongful conviction cases (type I) that took place between 1956 and 2017 were identified, coded and analyzed by two of the authors. Discrepancies in coding were resolved through discussion until consensus was reached.

A structured dataset was created, including the following variables: identity (or anonymized identifier), year of conviction, year of exoneration, type of offence, presence of scientific evidence, type of scientific error, and forensic discipline involved.

The classification of crime types used for this study has been simplified and does not correspond exactly to the charges. For example, the ‘murder’ category includes manslaughter as well as first- and second-degree murder, and all forms of sexual assault have been grouped together. In cases involving multiple charges, the most serious offence was retained.

The presence of scientific evidence was determined based on whether expert testimony or scientific findings were used during the investigation or trial.

Errors identified as having a scientific component were then classified according to whether they resulted from an analytical failure or an interpretation mistake. Analytical errors were defined as mistakes resulting from laboratory practices or technical procedures used during evidence analysis. Conversely, misinterpretation of scientific results refers to situations in which reliable analytical findings or observations were incorrectly interpreted, even in the absence of procedural or technical errors. In such cases, the intrinsic scientific value of the data was not questioned, and the analyses were reliable, but the meaning attributed to them was problematic. For example, an incorrect DNA profile resulting from laboratory contamination constitutes an analytical error, whereas an overstatement regarding the circumstances of transfer based on correct DNA findings constitutes an interpretative error.

The relevant scientific sub-discipline was then identified and categorised according to its forensic discipline. For example, the use of fibre or hair analysis was categorised under ‘micro-traces’.

These references only provide access to Canadian type I errors. The Canadian criminal procedure, by ensuring the admissibility and reliability of evidence and expert testimony, aims exclusively to limit such types of errors [30].

As we did not find any similar databases or references on type II errors in Canada, we turned to the Association d’Aide aux Victimes des Affaires Non-Élucidées (AVANE) [31], a French non-profit association that provides assistance to families of victims of unsolved cases in partnership with the French judicial system. AVANE chose three cases of interest for our exploratory approach. These cases were analyzed using a similar coding framework as for type I errors. Given the limited number of cases and the nature of the data, this component is intended as exploratory and illustrative rather than representative.

## 3 Results and discussion

### 3.1 Involvement of science

As stated before, wrongful convictions are complex and multifactorial phenomena involving numerous judicial actors, including scientific experts [1,22,27]. Among the 92 documented cases of wrongful convictions identified, we found that science was involved in 30 of those cases.

**Fig. 1:**
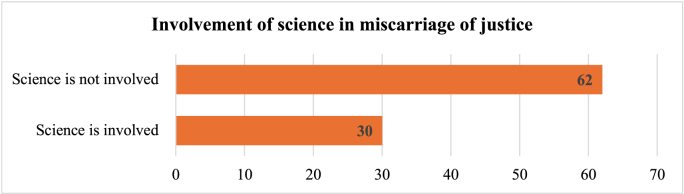
Involvement of science in wrongful convictions.

As shown in Fig. 1, these results suggest that among the cases studied, science is not primarily responsible for judicial errors. This observation is consistent with the scientific literature. For example, Collins and Jarvis reported that forensic science was involved in only 32 of the 283 American wrongful conviction cases studied [32], or approximately 11% of cases, a proportion significantly different from the 52% reported by the Innocence Project [33]. Huff, Rattner, and Sagarin reached similar conclusions: according to their study, less than 2% of miscarriages of justice are linked to scientific error [27].

**Fig. 2:**
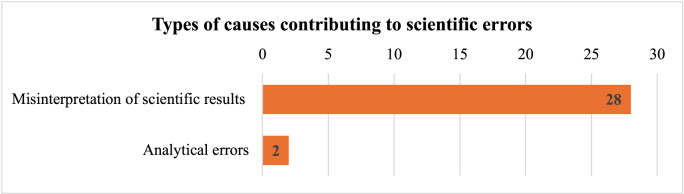
Type of causes contributing to scientific errors.

Although science is not involved in the majority of the 92 cases examined, it is nonetheless implicated in nearly a third of them. As shown in Fig. 2, among the 30 cases, we found that 97% (n = 28) resulted from the misinterpretation of scientific results, while only two cases (3%) were due to analytical errors. Notably, both analytical errors originated from the same laboratory, the *Mothersick Drug Testing Laboratory* at *Toronto's Hospital for Sick Children* [34].

Misinterpretation errors occur generally when results are exaggerated, taken out of context, or interpreted without considering scientific limitations, the broader context, or alternative hypotheses.

The case of Corey Robinson provides a clear illustration of this. In this case, the DNA analysis results linking Robinson to the victim were undisputed; all parties agreed that Robinson's DNA was indeed found under the victim's fingernails [35]. The source-level analysis was therefore reliable. The error occurred at the interpretative stage, when the expert extended the source-level conclusion to activity-level propositions regarding the circumstances of transfer. The three levels of proposition (source, activity, and offence), as proposed by Cook et al. [36], address distinct questions and should not be confused. In this case, the confusion between level of proposition, the source of the DNA, and the activity that causes the transfer, contributed to the wrongful conviction.

Such errors highlight the importance of the principle of hierarchy of propositions, which aims to ensure that experts do not exceed the scope of their expertise [37], emphasizing the separation between scientific analysis (experts) and inference to the facts (fact finders). In some analyzed cases, exceeding this framework, particularly when the expert comments on the probability of the cause, unduly influence the judicial decision. In the absence of critical analysis of the propositions, the fact-finder may then adopt the expert's interpretation as self-evident, thereby blurring the line between scientific expertise and judicial reasoning. This fallacy may be further reinforced by the semantic blurring between the concepts of probability and likelihood in common language, which are often used interchangeably despite their distinct meanings in a scientific context [38].

It would be useful to differentiate between errors in the interpretation of scientific results made by experts and those made by fact-finders. In some cases, it is the expert's erroneous or biased conclusions that contribute directly to judicial error. In others, it is the fact-finders, judges, or jury who place excessive value on the expert's opinion, sometimes going so far as to delegate their decision-making responsibility. As discussed by Champod and Vuille [39], when conclusions are presented as “scientific”, they tend to be perceived as objective, which may discourage critical scrutiny. This raises an important question regarding the extent to which fact-finders retain decision-making autonomy when expert opinions are not adequately contextualized or qualified.

### 3.2 Types of crime

The crimes most frequently observed among documented Canadian miscarriages of justice are violent crimes and crimes against the person, particularly murders (60 cases) and sexual assaults (19 cases). Crimes against the person are thus overrepresented among documented wrongful convictions when compared with their prevalence in general crime statistics. In 2024, Canada recorded approximately 778 homicides and 50,000 sexual assaults, compared with over 130,000 break-ins and more than 370,000 thefts under $5000 [40].

This disparity between the statistics reported by the Canadian government and the observations made in this research raises the possibility that documented wrongful convictions represent only a small subset of a larger phenomenon. It is plausible that other miscarriages of justice occur in cases deemed “less serious”, such as theft or burglary, but that they are neither contested nor publicized nor handled by organizations such as Innocence Canada. This is because such organizations focus primarily on cases involving heavy sentences or crimes of violence/against the person (such as murder or sexual assault). Furthermore, individuals accused of minor offenses may be more likely to plead guilty to avoid the costs, delays, and risks associated with trial, even when they are innocent [41]. As a result, such errors may remain largely invisible.

### 3.3 Discipline involved

**Fig. 3:**
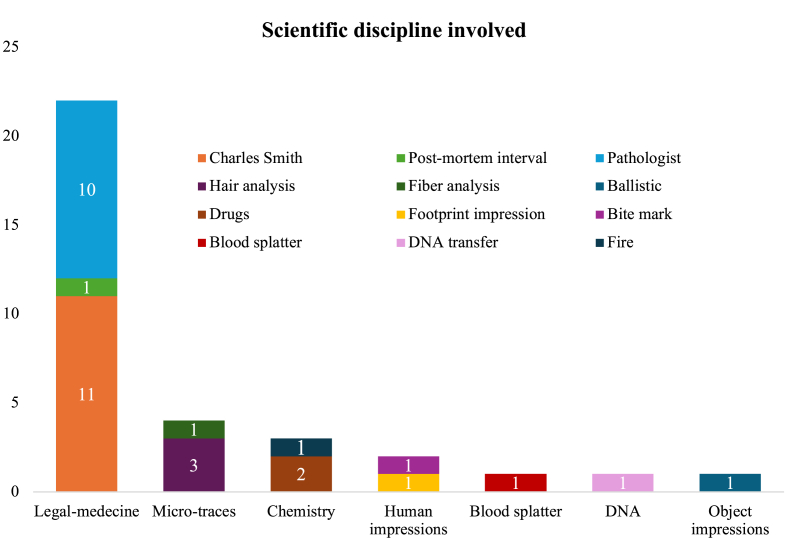
Number of wrongful conviction cases by scientific discipline implicated.

The 30 wrongful convictions involving science were categorised according to the scientific discipline implicated. As shown in Fig. 3, the most represented discipline among type I wrongful convictions involving science was legal medicine, which was involved in 22 out of 30 cases. It should be noted that multiple disciplines may be involved in a single wrongful conviction.

The overrepresentation of legal medicine can be partly explained by the fact that most recognized type I errors involve, as stated before, crimes against persons, cases of greater attention, and often requiring a pathologist's intervention (e.g., homicide, sexual assault, manslaughter). Therefore, the high prevalence of legal medicine in wrongful conviction statistics likely reflects the types of cases that receive sustained judicial and public scrutiny, rather than an inherent propensity for error within the discipline itself. In other words, pathologists' errors may be overrepresented because cases requiring the work of pathologists are also overrepresented in cases of miscarriage of justice.

This overrepresentation is further amplified by the presence of a problematic expert: Charles Smith, a pediatric pathologist from Ontario. The “Charles Smith case” even led to a provincial report on the practices of science in judicial settings (*The Inquiry into Pediatric Forensic Pathology in Ontario report* [42]). It illustrates how an expert's status can prevent critical scrutiny of their findings, despite a lack of scientific consensus or the use of unvalidated methods [42]. As a result, once such a pseudo-expert is exposed, numerous previously unnoticed errors resurface upon investigation, creating a “snowball effect” within the discipline. Thus, we observe that in this dataset, Smith is implicated in over 30% of wrongful convictions involving science, highlighting the systemic consequences of blind trust in expert authority.

Such uncritical acceptance of expert testimony can have procedural consequences. Challenging expert evidence in court can carry significant risks, including the possibility of harsher sentences if the challenge fails, which may discourage defense strategies and encourage guilty pleas [41]. This can also entail significant financial costs, as the defence often has to call upon independent experts, which is not necessarily within the means of all parties. This dynamic is further reinforced by a broader perception of scientific infallibility in judicial contexts, often described as the CSI effect [26].

In these circumstances, decision-makers may place excessive trust in expert conclusions, particularly when they lack the scientific knowledge necessary to assess uncertainty, methodological limitations, or alternative interpretations. This reliance can result in a *de facto* transfer of evaluative responsibility to experts, even when they are acting beyond the scope of validated scientific practice or outside the limits defined by admissibility standards such as those set out in R. v. Mohan [43].

The second most common involved discipline is micro-trace evidence, which includes hair and fiber analysis. Hair comparison evidence represents a particular case, as its probative value was historically overestimated despite limited scientific validation [44]. However, with the advent of DNA evidence in the 1990s, this type of identification evidence has virtually disappeared from modern courts. The use of this type of evidence has also been criticized in two other provincial public inquiries following miscarriages of justice: the *Report of the Commission of Inquiry into Certain Aspects of the Trial and Conviction of James Driskell* [44] and the *Commission on Proceedings Involving Guy Paul Morin* [45]. Both inquiries recommended that hair comparison evidence be used only for exclusionary purposes rather than inclusion, thus privileging the avoidance of type I errors over type II errors, echoing the principle that “*Il vaut mieux hasarder de sauver un coupable que de condamner un innocent.*” [46] (“*that ’tis much more Prudence to acquit two Persons, tho’ actually guilty, than to pass Sentence of Condemnation in one that is virtuous and innocent”* [47]).

Scientific evidence can also be instrumentalized during the investigation phase, particularly in cases affected by tunnel vision. In such situations, forensic findings are used primarily to confirm an existing hypothesis rather than to explore other possible explanations. The Clayton Johnson case illustrates this mechanism. Initially classified as an accidental fall, the death of Janice Johnson was reopened following rumors and an investigator's deep conviction in Clayton Johnson's guilt [48]. Subsequent changes in witness testimony, combined with a partial interpretation of bloodstain pattern analysis, contributed to his wrongful conviction. Although two RCMP experts expressed doubt regarding these conclusions, their dissenting opinions were not disclosed to the court [49,50]. Clayton Johnson was ultimately exonerated after spending 5 years in prison [51].

These considerations raise broader questions regarding the consequences of prioritizing the prevention of type I errors in criminal investigations, especially in a context where Canadian authorities regularly insist on the priority to support victims, hence to punish perpetrators [52,53].

It is also important to consider the relationship between type I and type II errors. In cases where a crime has actually been committed, a wrongful conviction (type I error) can simultaneously result in a type II error, namely the failure to identify and convict the true perpetrator. However, some wrongful convictions may occur in situations where no crime has been committed, for example in cases of accidents or misinterpreted events (like as in the Johnson case). In such cases, the corresponding concept of a type II error does not apply.

Therefore, observations made regarding type I errors may also be relevant to certain type II errors, particularly in cases where both stem from similar interpretative or systemic issues.

### 3.4 Type II cases

Due to confidentiality constraints, the level of detail that can be disclosed for these cases is limited. However, the available information allows for the identification of recurring patterns and mechanisms, which are explored here in an illustrative and exploratory manner rather than as a representative analysis.

The collaboration with AVANE revealed a mechanism contributing to type II errors in which forensic science plays a role. In the three cases examined (n = 3), investigators failed to recognize or adequately utilize relevant physical evidence identified or collected at the crime scene. These failures appear to result from a combination of technical limitations, resource constraints, and an underestimation of the potential of evidence to generate alternative hypotheses in contexts characterized by tunnel vision of the investigation.

For example, in all three cases, evidence identified at the scene were not examined in further detail because it was not initially considered relevant to the main hypothesis of the investigation. In one of the cases, potentially informative traces were collected but not analyzed due to resource constraints, which limited the number of analyses that could be performed per case. In another case, the traces were interpreted in a manner consistent with the initial hypothesis, without exploring alternative explanations that would have been more in line with witness testimony.

These observations suggest that type II errors can arise through several related mechanisms, such as failing to recognize the relevance of traces, failing to analyze the evidence collected, and selectively interpreting this evidence within a predefined investigative framework.

In all three cases studied, once the working hypothesis was established, investigative efforts focused primarily on collecting and analyzing evidence likely to confirm it, while other potentially informative traces were neglected or insufficiently exploited. This suggests that type II errors result not from a lack of evidence, but from an incomplete understanding and exploitation of available ones. However, to avoid anachronistic interpretations, this does not mean that mistakes were made. Rather, it supports the Sydney Declaration's emphasis that the interpretation and meaning of evidence depend on the context in which it is produced and evaluated.

### 3.5 Synthesis and implication

While acknowledging that some miscarriages of justice come from isolated human errors, these findings support that scientific miscarriages of justice predominantly come from deeper systemic vulnerabilities. These include structural deficiencies such as including inadequate scientific training among judicial actors, the instrumentalization of science to confirm preexisting hypotheses, the lack of knowledge regarding the information potential of traces, and insufficient rigor in evaluating methods and results.

Overall, these observations suggest that miscarriages of justice involving science cannot be adequately explained by isolated human or technical failures alone, let aside science only. Rather, they highlight structural vulnerabilities that affect how scientific evidence is produced, interpreted, understood, and used throughout both investigative and judicial processes [54].

## 4 Conclusion

This exploratory study examined the role of forensic science in wrongful convictions in Canada, complemented by three French unsolved cases, with a particular focus on the mechanisms through which scientific evidence contributes to both type I (false positives) and type II (false negatives) errors. Among the 92 documented Canadian cases analyzed, approximately a third involved scientific evidence. In the vast majority of these cases, the identified errors were interpretive rather than analytical, highlighting weaknesses not in laboratory performance but in the interpretation, management, and use of scientific results within the justice system.

The findings suggest that scientific miscarriages of justice stem from persistent systemic vulnerabilities, an observation also made in other study, like Sénéchal [55] who stated that errors in forensic science appear to be indicators of a judicial system prone to structural, epistemic and organisational biases; in other words, systemic shortcomings [55].

In type I errors, these vulnerabilities are characterized by an overestimation of expert authority and the probative value attributed to scientific conclusions. On the contrary, the French unsolved cases seem to support that an underestimation of the investigative potential of scientific evidence could partly explain type II errors, as its capacity to generate and nurture alternative hypotheses is not well exploited. In both cases, the underlying problem seems to lie in an insufficient understanding of the nature of evidence, its inferential role, and its epistemic limitations.

This study, based on a limited corpus of documented cases (n = 92 for type I errors and n = 3 for type II errors), is only exploratory, laying the ground for a research hypothesis underway. Nonetheless, the recurring patterns identified suggest that scientific miscarriages of justice are not isolated anomalies but a manifestation of systemic weaknesses in the management of forensic science within the Canadian legal system. Similar patterns have been reported in other jurisdictions; however, extending the findings of this study beyond the Canadian context would require further empirical investigation.

Overall, the findings of this exploratory research suggest a need for a better understanding of the limitations—but also of the inferential potential—of forensic evidence, both for scientists who work with such evidence and for other legal professionals in both the investigative and legal spheres. A better understanding of forensic evidence would necessarily impact the interpretation of scientific evidence, as well as its communication. It might also be worthwhile to review the procedures for managing forensic evidence, or even to consider integrating a forensic evidence specialist (forensic evidence expert) into investigations or court proceedings.

But addressing these issues requires re-centering forensic expertise on its object of study: the trace, a remnant of past activity, that supports reasoning through inferences [16].

## Ethical approval

Ethical approval was not required as this study is based on publicly available data.

## Funding

This research received no external funding.

## CRediT authorship contribution statement

**Sara-Jane Lécuyer:** Data curation, Formal analysis, Investigation, Writing – original draft, Writing – review & editing. **Keleiali'i Urima:** Data curation, Formal analysis, Investigation, Writing – original draft, Writing – review & editing. **Carole Sénéchal:** Supervision, Writing – review & editing. **Frank Crispino:** Conceptualization, Supervision, Writing – review & editing.

## Declaration of competing interest

The authors declare that they have no known competing financial interests or personal relationships that could have appeared to influence the work reported in this paper.
